# Modulatory Effect of Linoleic Acid During *Brucella abortus* 544 Infection in Murine Macrophage RAW264.7 Cells and Murine Model BALB/c Mice

**DOI:** 10.4014/jmb.1911.11037

**Published:** 2020-02-05

**Authors:** Alisha Wehdnesday Bernardo Reyes, Son Hai Vu, Tran Xuan Ngoc Huy, Wongi Min, Hu Jang Lee, Hong Hee Chang, John Hwa Lee, Suk Kim

**Affiliations:** 1 Institute of Animal Medicine, College of Veterinary Medicine, Gyeongsang National University, Jinju 52828, Republic of Korea; 2 College of Veterinary Medicine, Chonbuk National University, Iksan 54596, Republic of Korea

**Keywords:** *Brucella abortus*, cytokines, invasion, linoleic acid, spleen weight

## Abstract

In this study, we investigated the effects of linoleic acid (LA) treatment on *Brucella abortus* infection in professional phagocyte RAW264.7 cells, particularly during the pathogen’s invasion and intracellular growth in these cells, as well as in murine model BALB/c mice focusing on bacterial splenic proliferation and immunoregulatory activities. LA inhibited the growth of *Brucella* in a doseand time-dependent manner. The ability of the pathogen to enter the phagocytes was inhibited as was its survival within these cells. This was accompanied by increased nitrite accumulation in these cells at 24 h post-infection. The concentration of LA used in the present study did not affect the total body weight or liver function of the mice. During *Brucella* infection, the total splenic weight of these animals was not changed; rather, resistance to bacterial proliferation was enhanced in the spleen. Furthermore, mice treated with LA displayed elevated levels of IL-12 and IFN-γ but reduced levels of IL-10 during infection. The findings in this study showed the regulatory role of LA against *B. abortus* infection suggesting its potential use in designing intervention strategy for brucellosis.

## Introduction

*Brucella abortus* is a facultative intracellular bacterium that causes brucellosis which is primarily an animal disease leading to abortion and infertility in cattle, but exposure to infected animals or animal by-products can cause disease in humans causing undulant fever, debilitating arthritis, endocarditis and meningitis [1, 2]. Brucellae are readily phagocytosed by polymorphonuclear cells and macrophages, and replicate intracellularly while suppressing host immune response and evading the action of the infected cells – promoting chronicity of infection [3, 4, 5]. No *Brucella* vaccines are available for humans and although conventional antibiotic regimens are applicable for infected patients that may last for several months, these are not always completely effective and relapses are still observed [6]. Furthermore, even in cattle, most successful adult vaccine (S19) results in orchitis, prolonged infection and abortion complications, and serological tests used for disease diagnosis are often found to be misleading [7, 8].

In our unpublished data, we performed metabolome profiling of plasma samples from *B. abortus*-infected mice at 10, 30 and 60 days post-infection and several polyunsaturated fatty acids (PUFA) were identified as potential plasma biomarkers for diagnosing brucellosis. New tools for diagnosis and new biomarkers could hold keys to evaluate both pathogen and host response to infection. Constant exposure to various pathogenic organisms endowed hosts with several endogenous anti-microbial compounds including interferon, cytokines, free radicals, etc., but little attention has been paid to lipids given the fact that they are present in all tissues of the body [9]. Several studies on antimicrobial effect of PUFAs have been reported against the growth of fungi, protozoan, viruses and various types of bacteria such as methicillin-resistant *Staphylococcus aureus*, *Helicobacter pylori* and *Mycobacteria* [10, 11]. Particularly, the antibacterial actions that are usually attributed to PUFAs include linoleic acid (LA) [10]. LA is a constituent of acylglycosyl ceramides with a physiological role in maintaining the water permeability barrier of the skin, and is converted mainly to arachidonic acid which may lead to increased production of downstream pro-inflammatory metabolites [12, 13]. Furthermore, LA was one of the most abundant compounds found in Korean red ginseng oil (KRGO) through phytochemical analysis, as we previously reported [14]. To our knowledge, no reports have documented the action of LA on *B. abortus* or taken into consideration the regulatory roles associated with LA, so we investigated its effects on the course of brucellosis in murine macrophages and in a murine model.

## Materials and Methods

### Ethics Statement

The animal procedures performed in this study were approved by the Animal Ethical Committee of Chonbuk National University (Authorization Number CBNU-2018-119).

### Linoleic Acid (LA) Preparation

LA (molecular weight 280.45 g/mol; Sigma-Aldrich, USA) was dissolved in absolute ethanol (1 M) and further diluted in sterile phosphate-buffered saline solution (PBS, pH 7.4) containing 0.1% bovine serum albumin (BSA, GenDEPOT, USA).

### Bacteria

A smooth, virulent *Brucella abortus* biovar 1 strain *B. abortus* 544 (ATCC 23448) was maintained on Brucella agar (1.5% agar) (Becton Dickinson, USA) and grown in broth at 37°C with shaking until the stationary phase. The bacteria were suspended in PBS and the number of viable bacteria was measured by plating serial dilutions on Brucella agar plates.

### Cells

RAW 264.7 cells (ATCC TIB7-1, USA) were maintained at 37°C in 5% CO_2_ in RPMI 1640 containing 10% heat-inactivated fetal bovine serum (FBS), 2 mM L-glutamine, 100 U/ml penicillin, and 100 µg/ml streptomycin (all provided by Gibco, USA) and were seeded (1 × 10^5^ cells/ml in 96-well plates; 1 × 10^6^ cells/ml in 6-well plates) in tissue culture plates overnight. Cells were incubated in fresh medium without antibiotics prior to all bacterial infection assays.

### Cytotoxicity Assay

RAW264.7 cells were prepared in a 96-well plate overnight and then incubated at different concentrations of LA (0, 10, 20, 50, 100, 200, 500 µM) for 48 h. Cytotoxicity analysis was performed using MTT assay as previously reported [14]. The control used contains 0.1% ethanol and 0.1% BSA in fresh medium without antibiotics in all in vitro assays.

### Bactericidal Assay

Bacteria were grown to stationary phase and diluted using PBS (2 × 10^4^ colony forming units, CFU). A 10 µl bacterial solution was added to different concentrations of LA (0, 10, 50, 100, 500 µM) for 0, 2, 24, and 48 h. Bactericidal analysis was performed using CFU counts as previously reported [14].

### Nitrite Assay

RAW264.76 cells were prepared in a 96-well plate overnight and then incubated with or without LA (10 µM) for at least 4 h. The cells were infected with *B. abortus* at a multiplicity of infection (MOI) of 100 for 1 h, washed with PBS and then incubated in fresh medium containing gentamicin (30 µg/ml) with or without LA as previously described [14]. Nitrite accumulation was measured using Griess reagent (Promega, USA) at 2, 24, and 48 h post-infection according to manufacturer’s instruction.

### Infection Assay

For internalization assay, RAW264.7 cells were prepared in a 96-well plate overnight and incubated with or without LA for at least 4 h as previously described [14]. The cells were washed and then infected with *B. abortus* at MOI of 100 for 0 and 30 min. After infection, the medium was changed to fresh medium containing gentamicin and incubated further for 30 min. After washing, cells were lysed using distilled water and then diluted using PBS. The diluent was then plated onto Brucella agar and incubated for 3 d. Bacterial internalization efficiency was evaluated by counting colony forming units (CFUs). For intracellular growth assay, preparation, infection and plating of cells, and analysis of intracellular growth efficiency were the same as that of the internalization assay as previously described [14]. After infection for 1 h, the cells were washed and then incubated in fresh medium containing gentamicin (30 µg/ml) with or without LA for 0, 2, 24, and 48 h. Culture supernatants were collected to analyze cytokine levels during *B. abortus* infection in macrophages.

### Animal Experiment

Eight-week-old, pathogen-free female BALB/c mice (Samtako Bio Co. Ltd., Korea) acclimatized for one week were randomly divided into four groups of five mice. The groups were further subdivided into non-infected and *Brucella*-infected groups. A gavage needle was used to orally give 100 µl of LA (10 µM) or vehicle (0.1% ethanol and 0.1% BSA in PBS) for three days prior to infection until 14 days post-infection as previously reported [14]. For the infected groups, the mice were intraperitoneally injected with *B. abortus* (2 × 10^4^ CFU in 100 µl PBS). At 3 days post-infection, blood was collected via tail vein and at 14 days post-infection, mice were sacrificed, blood was collected from the heart and the spleens were collected. The spleens were weighed and a part was homogenized, serially diluted in PBS and then plated onto Brucella agar to determine the number of CFUs in the spleens of each group.

### ELISA

Serum alanine aminotransferase 1 (ALT) concentration was quantified using an ALT (Mouse) ELISA Kit (BioVision Inc., USA) to monitor hepatocellular injury during LA treatment according to manufacturer’s instruction.

### Flow Cytometry

Culture supernatants and serum samples were processed to measure the different levels of cytokines involved in the course of brucellosis including IL-12p70, TNF, IFN-γ, MCP-1, IL-10, and IL-6 using a Cytometric Bead Array (CBA) mouse inflammation kit (BD Biosciences, USA) according to manufacturer’s instruction.

### Statistical Analysis

The data are expressed as the mean ± standard deviation (SD) of triplicate samples from at least three independent experiments. Student’s t-test was used to make statistical comparisons between groups using GraphPad InStat software version 3 (GraphPad Software, Inc., USA). Differences of *p* < 0.05 were considered significantly different.

## Results

### Effect of LA on Viability of RAW264.7 Cells and Survival of *B. abortus*

**Fig. 1:**
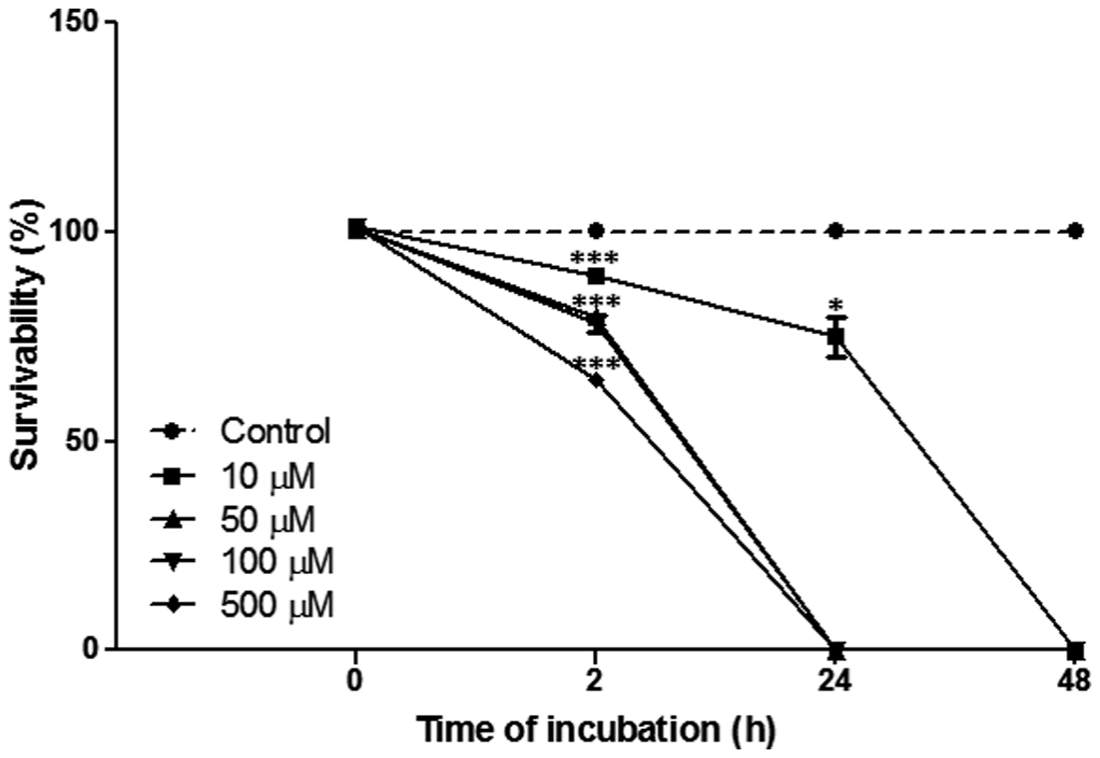
The bactericidal effect of the different concentrations of LA (0, 10, 50, 100, and 500 μM) against *B. abortus* incubated for 0, 2, 24, and 48 h. Data represent the mean ± SD of at least three replicates. Notes: *p < 0.05, ***p < 0.001, compared with control group.

Decreased OD values were observed in RAW264.7 cells treated with LA at concentrations of 20, 50, 100, 200, and 500 µM. OD values did not change in cells incubated at 10 µM (data not shown) compared to untreated controls, hence treatment with LA was applied at a concentration of 10 µM in the subsequent experiments. On the other hand, bacterial cells incubated with various concentrations of LA (10, 50, 100, and 500 µM) significantly inhibited the growth of *B. abortus* at 2 h post-incubation compared to untreated controls (Fig. 1). Bacterial growth was completely inhibited at all concentrations tested at 48 h post-incubation indicating that LA has a bactericidal effect against *B. abortus*.

### Effect of LA on Internalization and Intracellular Survival of *B. abortus*

**Fig. 2:**
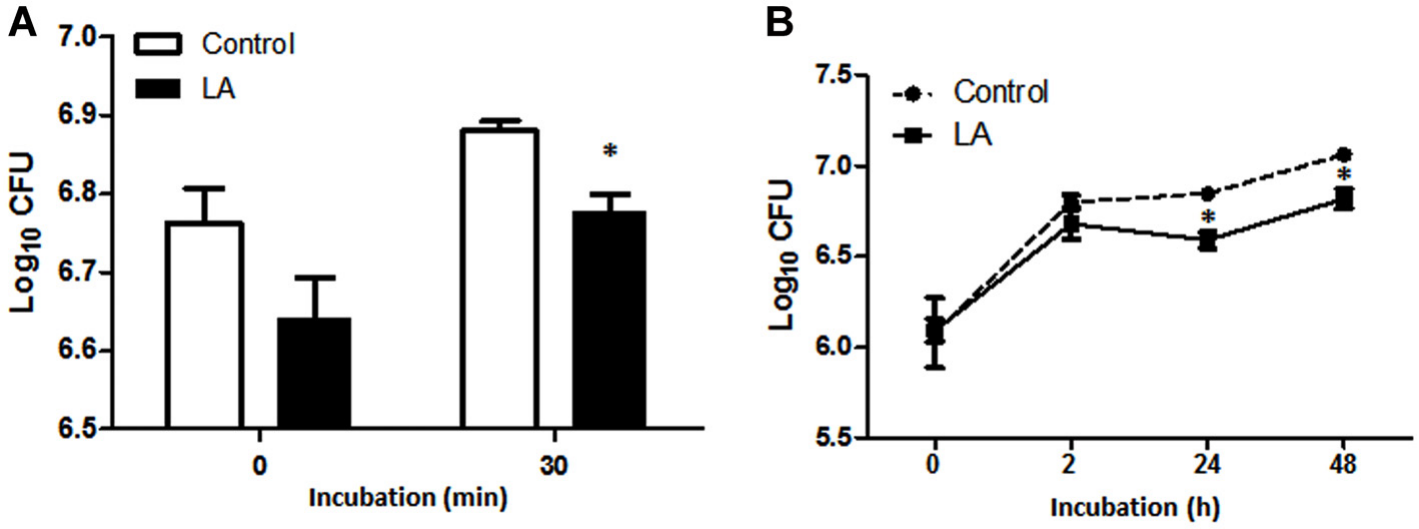
The inhibitory effect of LA on (A) internalization and (B) intracellular growth efficiency of *B. abortus* in RAW264.7 cells incubated at indicated times. Data represent the mean ± SD of at least three replicates. Note: *p < 0.05, compared with control group.

RAW264.7 cells were pretreated with LA for at least 4 h prior to *B. abortus* infection to determine the effect of LA on the ability of the pathogen to invade macrophages. At 0 min post-infection, the number of internalized bacteria was reduced in LA-treated cells but the difference was not significant compared to untreated controls (Fig. 2A). This was observed to markedly decrease at 30 min (*p* < 0.05) post-infection suggesting that LA could negatively affect bacterial invasion of *B. abortus* into RAW264.7 cells. On the other hand, the number of bacteria that survived within RAW264.7 cells was significantly attenuated at 24 (*p* < 0.05) and 48 h (*p* < 0.05) post-infection treatment with LA (Fig. 2B) compared to untreated controls. Taken together, LA treatment could interfere in the internalization as well as intracellular survival of *B. abortus* in macrophages.

### Effect of LA on Nitrite Production in RAW264.7 Cells

**Fig. 3:**
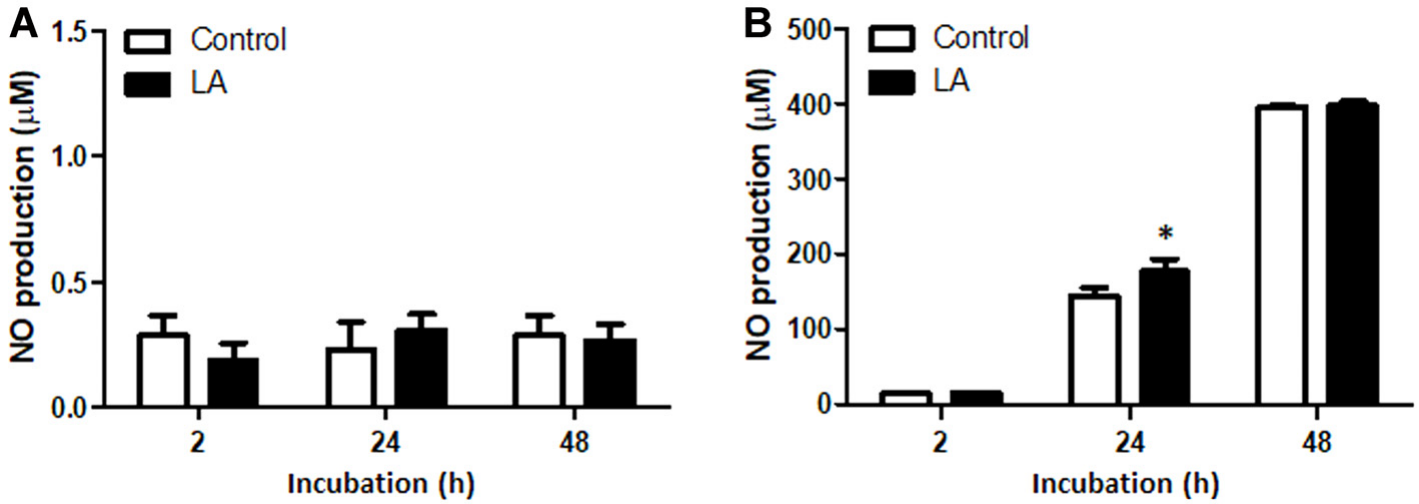
The effect of LA on nitrite accumulation in RAW264.7 cells during (A) without infection and (B) *B. abortus* infection at indicated times. Data represent the mean ± SD of at least three replicates. Note: *p < 0.05, compared with control group.

RAW264.7 cells were pretreated with LA for at least 4 h and then infected with *B. abortus* for 1 h. The cells were subsequently incubated with fresh medium containing LA and gentamicin for 2, 24 and 48 h. Nitrite accumulation was measured using Griess assay as an indicator of nitric oxide (NO) and NO is known as an important effector molecule for the clearance of *B. abortus*. Here, the concentration of nitrite did not significantly change during normal condition or without infection until 48 h (Fig. 3A). However, nitrite production was observed to significantly increase at 24 h (*p* < 0.05) post-infection treatment with LA compared to untreated controls (Fig. 3B).

### Effect of LA on Cytokine Production in RAW264.7 Cells

**Fig. 4:**
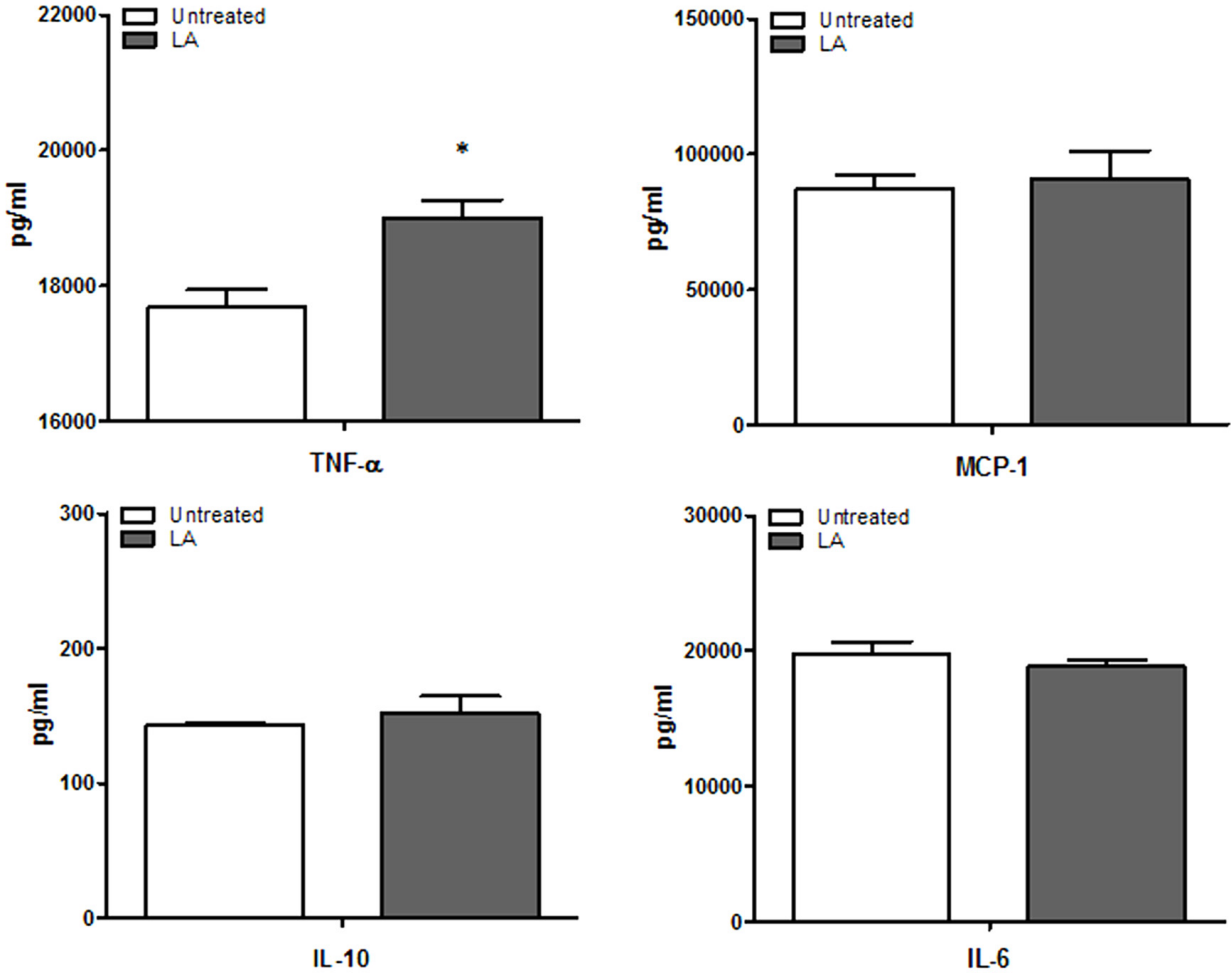
The effect of LA on cytokine production in RAW264.7 cells during *B. abortus* infection. Data represent the mean ± SD of at least three replicates. Note: *p < 0.05, compared with control group.

RAW264.7 cells were infected with *B. abortus* for 1 h and the cell culture supernatants were collected at 48 h post-infection for cytokine analysis using CBA. LA treatment in cells showed increased production levels of TNF-α (*p* < 0.05) as compared to untreated controls (Fig. 4). Levels of IL-12 and IFN-γ were not detected in untreated or treated cells.

### Effect of LA on *B. abortus* Infection in Mice

**Fig. 5:**
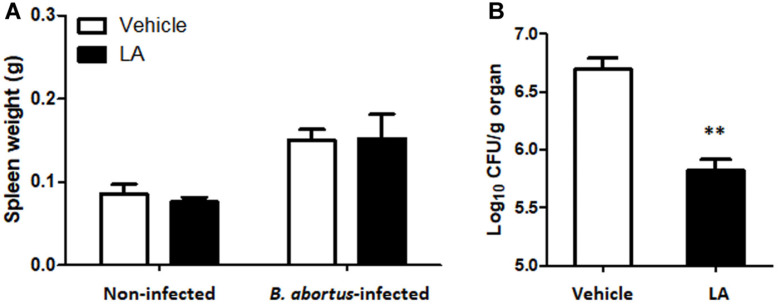
The effect of LA on (A) spleen weight and (B) bacterial splenic proliferation during *B. abortus* infection in BALB/c mice. Data represent the mean ± SD of five mice. Note: **p < 0.01, compared with control group.

The mice were observed for any clinical symptoms during the entire treatment period. Body weight and serum ALT concentrations were checked at the end of the experiment and showed no differences between treated and untreated control groups without *B. abortus* infection (data not shown). Liver and spleen are the most conspicuously infected organs but spleens showed higher number of CFU per gram of organ during the course of infection in mice, hence the preferred target organ to study *Brucella* infections in this animal model [15]. On the other hand, total weight of the spleens of the treated group was not significantly different from untreated group both in the uninfected and infected groups (Fig. 5A). However, the number of log CFU in the spleens recovered from treated group (*p* < 0.01) was significantly lower than untreated group (Fig. 5B).

### Effect of LA on Cytokine Production During *B. abortus* Infection in Mice

**Fig. 6:**
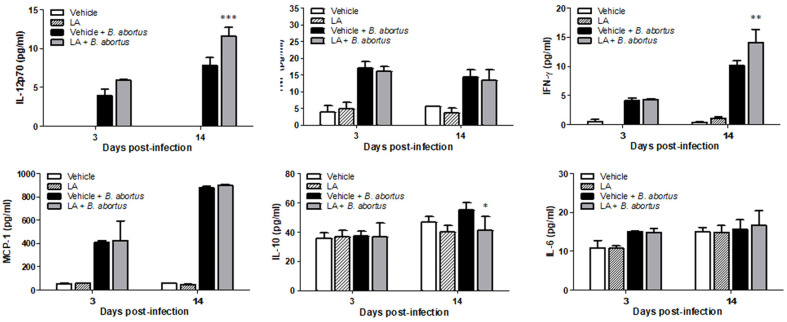
The effect of LA on the production of serum cytokines in BALB/c mice at 3 and 14 d post-infection. Data are presented as the means ± SD for each group. Notes: *p < 0.05, **p < 0.01, compared with *B. abortus*-infected group.

In the uninfected groups, no significant differences in the serum level of cytokines were observed between LA-treated and untreated groups at 3 and 14 d post-infection. On the other hand, in the *B. abortus*-infected groups, elevated levels of IL-12 (*p* < 0.001) and IFN-γ (*p* < 0.01) were observed in LA-treated mice as compared to control at 14 d post-infection (Fig. 6). However, a significantly reduced level of IL-10 (*p* < 0.05) was observed in treated group at 14 d post-infection (Fig. 6).

## Discussion

PUFAs have been suggested to function as endogenous anti-bacterial, anti-fungal, anti-viral, anti-parasitic and immunomodulating agents [9]. It was also proposed that PUFAs hold inhibitory action against bacterial growth via cell membrane disruption [11]. Dilika *et al*. [16] isolated LA from the dichloromethane extract of *Helichrysum pedunculatum* leaves and reported that it inhibited the growth of all gram-positive bacterial species tested but was inactive against gram-negative species such as *Enterobacter cloacae*, *Escherichia coli*, *Klebsiella pneumoniae*, *Pseudomonas aeruginosa* and *Serratia marcescens*. Interestingly, LA in the present study successfully inhibited the growth of *B. abortus* in a dose- and time-dependent manner. Zheng *et al*. [10] reported that LA inhibited bacterial enoyl-acyl carrier protein reductase (FabI) that correlated with the inhibition of fatty acid biosynthesis and antibacterial activity. Fatty acid synthesis, of which FabI is an essential component, is necessary in the production of a number of lipid-containing components such as the cell membranes. Similarly, Peng *et al*. [17] showed that the total LA production by *mcra*-inserted *Lactobacillus casei* was raised to 21-fold and the cell-free culture supernatants from this organism completely excluded survival of *Salmonella* Typhimurium and enterohaemorrhagic *E. coli* (EHEC) at 72 h and 48 h, respectively, and as compared to the wild-type bacterium, exhibited more effectiveness in lowering hydrophobicity and autoaggregation activities with more intensified cell membrane disruption in these pathogens. Therefore, there is a possibility that the antibacterial action of LA could be due to inhibition of bacterial fatty acid synthesis although this remains to be proven.

*B. abortus* is a stealthy intracellular pathogen of animals and humans that can circumvent immune response and replicate within macrophages for survival and establishment of chronic infections [18]. Here, LA treatment negatively affected internalization of *Brucella* into RAW264.7 cells while also impeding the bacterial survivability inside these cells accompanied with increased nitrite accumulation at 24 h post-infection. We previously reported that extraction of fermented rice bran mixture extract (RBE) using ethanol identified LA in its primary chemical composition, and RBE showed inhibitory effect against the uptake of *B. abortus* in RAW264.7 and HeLa cells but did not alter the intracellular growth of the pathogen in these cells [19]. However, we also reported the inhibitory mechanisms of KRGO against phagocytic and intracellular survival of *Brucella* in RAW264.7 cells [14] which could be contributed by LA since this compound was found to be one of the most abundant components identified in the phytochemical analysis of KRGO. NO, on the other hand, is known as the effector molecule against various intracellular pathogens and reported to accelerate killing of intracellular *B. abortus* in macrophages although not to completion during the first 24 h of infection [20, 21]. Liang and Akaike [22] showed that the combination of LA and IFN-γ induced NO synthesis in primary parenchymal hepatocytes from mice. In a study done by Babu *et al*.[23], all the fatty acids they tested including LA were taken up by a chicken macrophage-like cell line, HD11, but did not affect uptake of green fluorescent protein-labeled *Salmonella* Typhimurium in these cells. Furthermore, clearance of *Salmonella* was significantly higher with LA but was not associated with increased NO production by HD11 cells. There is a possibility that LA in the present study was taken up by RAW264.7 cells that contributed in the reduced susceptibility of these cells to *Brucella* infection due to its bactericidal effect. However, further confirmation is needed to demonstrate the direct effect of LA against the bacterium during the infection in macrophages. On the other hand, elevated TNF-α level in LA-treated murine macrophages might contribute in the control of *Brucella* infection in these cells since experimental evidence revealed the beneficial role of this cytokine in the reduction of *Brucella* spp. replication in human macrophages as well as its direct contribution against *Brucella* infection in mice [7].

Cell-mediated immunity and macrophage activation, both controlled by cytokine production during infection, are associated with host resistance to intracellular parasites [20]. We therefore further investigated the immunomodulatory activities of LA on *B. abortus* infection in vivo using a mouse model. Changhua *et al*. [24] reported that dietary supplementation of conjugated LA in LPS-injected pigs alleviated growth depression and prevented elevations in plasma concentrations of pro-inflammatory cytokines IL-6 and TNF but enhanced plasma level of anti-inflammatory IL-10. In a study done by He *et al*. [25], dietary supplementation of conjugated LA enhanced immune response in broiler chicks. Here, serum IL-6 and TNF levels in mice were not affected, however IL-10 was attenuated at 14 d post-infection. *Brucella* infection in mice is known to activate Type1 (Th1) cellular immune response promoting bacterial clearance under the control of TNF, IFN-γ and IL-12 [26]. LA-treated mice in the present study also displayed elevated IL-12 and IFN-γ. Depletion of endogenous IL-12 prior to *B. abortus* infection in mice reduced splenomegaly and significantly enhanced *Brucella* infection [27]. IFN-γ-producing T cells play a key role in the protective immunity against *B. abortus* and IL-12 is reported to possess a profound effect on the stimulation of CD4+ T cells and NK cells in producing IFN-γ that overall contribute in the control of infection [27]. Lack of IL-12 production in mice has been reported to participate in the progress of *Brucella* infection [28]. IFN-γ was found to be lower in activated peripheral blood mononuclear cells from piglets fed with conjugated LA while no differences were observed on the level of TNF and IL-10 [29]. The reported mechanism by which IFN-γ enhances resistance to *Brucella* infection in vitro is largely mediated by the anti-Brucella activity of activated macrophages with enhanced production of reactive oxygen intermediates [27]. Furthermore, IFN-γ contributes in the control of intracellular microbial pathogens and has been demonstrated to reduce the number of *B. abortus* by 10-fold in BALB/c mice supplemented with recombinant IFN-γ [30]. IL-10, on the other hand, has been investigated to modulate pro-inflammatory immune response to *B. abortus* infection and lack of this cytokine leads to *B. abortus* clearance in mice [31]. It has been suggested that IL-10 downregulates immune response to *B. abortus* even in the presence of IFN-γ in BALB/c mice, and inhibits the anti-Brucella effector functions of macrophages and the production of the protective IFN-γ by spleen cells [32]. Although the splenic weight did not change during LA treatment, the number of *Brucella* significantly reduced, which can be attributed to the enhanced production of pro-inflammatory cytokines although the bactericidal effect of LA against the pathogen during the course of the infection cannot be ruled out as observed in the in vitro analysis. However, it should be noted that high intake of LA in a diet deficient in other PUFAs can lead to high tissue production of prostaglandin E2 which in turn inhibits proliferation and cytokine production of Th1 cells [33]. Taken together, the data presented in this study showed the beneficial effect of LA treatment against *Brucella* infection suggesting its application in designing intervention strategy against brucellosis.

## References

[1] Negron ME, Kharod GA, Bower WA, Walke H (2019). Notes from the field: human *Brucella abortus* RB51 infections caused by consumption of unpasteurized domestic dairy products - United States, 2017-2019. MMWR Morb. Mortal. Wkly. Rep..

[2] Guimarães ES, Gomes MTR, Campos PC, Mansur DS, dos Santos AA, Harms J (2019). *Brucella abortus* cyclic dinucleotides trigger STING-dependent unfolded protein response that favors bacterial replication. J. Immunol..

[3] de Figueiedo P, Ficht TA, Rice-Ficht A, Rossetti CA, Adams LG (2015). Pathogenesis and immunobiology of brucellosis. Am. J. Pathol..

[4] Barquero-Calvo E, Mora-Cartin R, Arce-Gorvel V, de Diego JL, Chacon-Diaz C, Chaves-Olarte E (2015). *Brucella abortus* induces the premature death of human neutrophils through the action of its lipopolysaccharide. PLoS Pathog..

[5] Bosilkovski M (2019). Brucellosis: Microbiology, Epidemiology, and Pathogenesis.

[6] Yang X, Skyberg JA, Cao L, Clapp B, Thornburg T, Pascual DW (2013). Progress in *Brucella* vaccine development. Front. Biol. (Beijing).

[7] Dorneles EMS, Sriranganathan N, Lage AP (2015). Recent advances in *Brucella abortus* vaccines. Vet. Res..

[8] Khan MZ, Zahoor M (2018). An overview of brucellosis in cattle and humans, and its serological and molecular diagnosis in control strategies. Trop. Med. Infect. Dis..

[9] Das UN (2018). Arachidonic acid and other unsaturated fatty acids and some of their metabolites function as endogenous antimicrobial molecules: a review. J. Adv. Res..

[10] Zheng CJ, Yoo JS, Lee TG, Cho HY, Kim YH, Kim WG (2005). Fatty acid synthesis is a target for antibacterial activity of unsaturated fatty acids. FEBS Lett..

[11] Correia M, Michel V, Matos AA, Carvalho P, Oliveira MJ, Ferreira R (2012). Docosahexaenoic acid inhibits *Helicobacter pylori* growth *in vitro* and mice gastric mucosa colonization. PLoS One..

[12] Sanders TAB (2016). Functional Dietary Lipids: Food Formulation, Consumer Issues and Innovation for Health.

[13] Lankinen MA, Fauland A, Shimizu B, Agren J, Wheelock CE, Laakso M (2019). Inflammatory response to dietary linoleic acid depends on *FADS1* genotype. Am. J. Clin. Nutr..

[14] Reyes AWB, Hop HT, Arayan LT, Huy TXN, Park SJ, Kim KD (2017). The host immune enhancing agent Korean red ginseng oil successfully attenuates *Brucella abortus* infection in a murine model. J. Ethnopharmacol..

[15] Grillo MJ, Blasco JM, Gorvel JP, Moriyon I, Moreno E (2012). What have we learned from brucellosis in the mouse model?. Vet. Res..

[16] Dilika F, Bremner PD, Meyer JJ (2000). Antibacterial activity of linoleic and oleic acids isolated from *Helichrysum pedunculatum*: a plant used during circumcision rites. Fitoterapia.

[17] Peng M, Tabashsum Z, Patel P, Bernhardt C, Biswas D (2018). Linoleic acids overproducing *Lactobacillus casei* limits growth, survival, and virulence of *Salmonella* Typhimurium and enterohaemorrhagic *Escherichia coli*. Front. Microbiol..

[18] Gutierrez-Jimenez C, Mora-Cartin R, Altamirano-Silva P, Chacon-Diaz C, Chaves-Olarte E, Moreno E, Barquero-Calvo E (2019). Neutrophils as trojan horse vehicles for *Brucella abortus* macrophage infection. Front. Immunol..

[19] Hop HT, Arayan LT, Reyes AWB, Huy TXN, Baek EJ, Min WG (2017). Inhibitory effect of the ethanol extract of a rice bran mixture comprising *Angelica gigas*, *Cnidium officinale*, *Artemisia princeps*, and *Camellia sinensis* on *Brucella abortus* uptake by professional and nonprofessional phagocytes. J. Microbiol. Biotechnol..

[20] Gross A, Spiesser S, Terraza A, Rouot B, Caron E, Dornand J (1998). Expression and bactericidal activity of nitric oxide synthase in *Brucella suis*-infected murine macrophages. Infect. Immun..

[21] Wang M, Qureshi N, Soeurt N, Splitter G (2001). High levels of nitric oxide production decrease early but increase late survival of *Brucella abortus* in macrophages. Microb. Pathog..

[22] Liang JF, Akaike T (1998). Protective effect of linoleic acid on IFN-γ-induced cellular injury in primary culture hepatocytes. J. Biochem..

[23] Babu U, Wiesenfeld P, Gaines D, Raybourne RB (2009). Effect of long chain fatty acids on *Salmonella* killing, superoxide and nitric oxide production by chicken macrophages. Int. J. Food Microbiol..

[24] Changhua L, Yindong Y, Defa L, Lidan Z, Shiyan Q, Jianjun X (2005). Conjugated linoleic acid attenuates the production and gene expression of proinflammatory cytokines in weaned pigs challenged with lipopolysaccharide. J. Nutr..

[25] He X, Zhang H, Yang X, Zhang S, Dai Q, Xiao W (2007). Modulation of immune function by conjugated linoleic acid in chickens. Food Agr. Immunol..

[26] Dornand J, Gross A, Lafont V, Liautard J, Oliaro J, Liautard JP (2002). The innate immune response against *Brucella* in humans. Vet. Microbiol..

[27] Zhan Y, Cheers C (1995). Endogenous interleukin-12 is involved in resistance to *Brucella abortus* infection. Infect. Immun..

[28] Macedo GC, Magnani DM, Carvalho NB, Bruna-Romero O, Gazzinelli RT, Oliveira SC (2008). Central role of MyD88-dependent dendritic cell maturation and proinflammatory cytokine production to control *Brucella abortus* infection. J. Immunol..

[29] Malovrh T, Kompan L, Juntes P, Wraber B, Spindler-Vesel A, Kompan D (2009). Influence of conjugated linoleic acid on the porcine immune response and morbidity: a randomized controlled trial. Lipids Health Dis..

[30] Murphy EA, Sathiyaseelan J, Parent MA, Zou B, Baldwin CL (2001). Interferon-g is crucial for surviving a *Brucella abortus* infection in both resistant C57BL/6 and susceptible BALB/c mice. Immunol..

[31] Corsetti PP, de Almeida LA, Carvalho NB, Azevedo V, Silva TMA, Teixeira HC (2013). Lack of endogenous IL-10 enhances production of proinflammatory cytokines and leads to *Brucella abortus* clearance in mice. PLoS One.

[32] Fernandes DM, Baldwin CL (1995). Interleukin-10 downregulates protective immunity to *Brucella abortus*. Infect. Immun..

[33] Sammon AM (1999). Dietary linoleic acid, immune inhibition and disease. Postgrad. Med. J..

